# *Cryptosporidium parvum* regulates HCT-8 cell autophagy to facilitate survival via inhibiting miR-26a and promoting miR-30a expression

**DOI:** 10.1186/s13071-022-05606-y

**Published:** 2022-12-15

**Authors:** Heng Jiang, Xu Zhang, Xin Li, Xiaocen Wang, Nan Zhang, Pengtao Gong, Xichen Zhang, Yanhui Yu, Jianhua Li

**Affiliations:** 1 State Key Laboratory for Zoonotic Diseases, Key Laboratory for Zoonosis Research of the Ministry of Education, Institute of Zoonosis and College of Veterinary Medicine, Jilin University, Changchun, 130062 China; 2 The Second Hospital of Jilin University, Changchun, 130021 Jilin China

**Keywords:** *Cryptosporidium parvum*, microRNAs, MAPK signaling, Autophagy, Parasite proliferation

## Abstract

**Background:**

*Cryptosporidium parvum* is an important zoonotic parasite, which not only causes economic losses in animal husbandry but also harms human health. Due to the lack of effective measures for prevention and treatment, it is important to understand the pathogenesis and survival mechanism of *C. parvum*. Autophagy is an important mechanism of host cells against parasite infection through key regulatory factors such as microRNAs and MAPK pathways. However, the regulatory effect of *C. parvum* on autophagy has not been reported. Here, we demonstrated that *C. parvum* manipulated autophagy through host cellular miR-26a, miR-30a, ERK signaling and P38 signaling for parasite survival.

**Methods:**

The expression of Beclin1, p62, LC3, ERK and P38 was detected using western blotting in HCT-8 cells infected with *C. parvum* as well as treated with miR-26a-mimic, miR-30a-mimic, miR-26a-mimic or miR-30a-inhibitor post *C. parvum* infection. The qPCR was used to detect the expression of miR-26a and miR-30a and the number of *C. parvum* in HCT-8 cells. Besides, the accumulation of autophagosomes was examined using immunofluorescence.

**Results:**

The expression of Beclin1 and p62 was increased, whereas LC3 expression was increased initially at 0–8 h but decreased at 12 h and then increased again in *C. parvum*-infected cells. *C. parvum* inhibited miR-26a-mimic-induced miR-26a but promoted miR-30a-mimic-induced miR-30a expression. Suppressing miR-30a resulted in increased expression of LC3 and Beclin1. However, upregulation of miR-26a reduced ERK/P38 phosphorylation, and inhibiting ERK/P38 signaling promoted Beclin1 and LC3 while reducing p62 expression. Treatment with miR-26a-mimic, autophagy inducer or ERK/P38 signaling inhibitors reduced but treatment with autophagy inhibitor or miR-30a-mimic increased parasite number.

**Conclusions:**

The study found that *C. parvum* could regulate autophagy by inhibiting miR-26a and promoting miR-30a expression to facilitate the proliferation of parasites. These results revealed a new mechanism for the interaction of *C. parvum* with host cells.

**Graphical Abstract:**

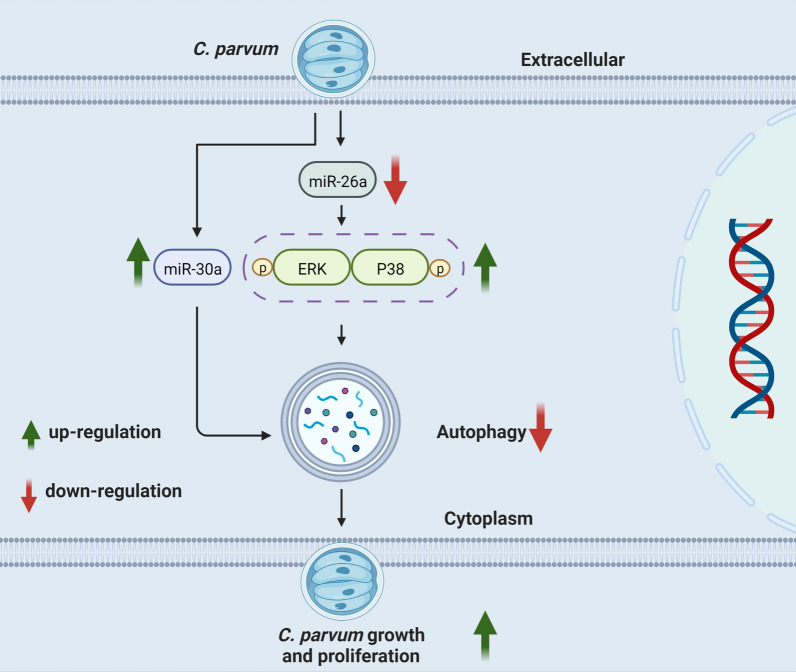

**Supplementary Information:**

The online version contains supplementary material available at 10.1186/s13071-022-05606-y.

## Background

*Cryptosporidium* is considered to be the second major cause of diarrhea and death in children [1, 2]. *Cryptosporidium* has thus far 44 recognized species and more than 120 genotypes, of which 17 species and 4 genotypes are zoonotic [3, 4]. *Cryptosporidium parvum* infects the gastrointestinal tract of animals and humans [6]. The main clinical manifestation of *C. parvum* infection is watery diarrhea, which usually occurs 3–4 days after infection of oocysts, lasting for about 1–2 weeks [5]. In addition, loss of appetite, drowsiness, dehydration and even death can also be observed. At present, there are no vaccines for *Cryptosporidium*; the drug commonly used for cryptosporidiosis is nitazoxanide, with little therapeutic effect for cryptosporidiosis patients with immune deficiency [7]. Mutant kinase inhibitors of calcium-dependent protein kinase 1 (CDPK1) and phosphatidylinositol-4-OH kinase inhibitors are potential new drugs to treat cryptosporidiosis [8–10]. An in-depth understanding of the underlying pathogenic mechanisms of *Cryptosporidium* will be helpful to discover new potential therapeutic targets.

Autophagy is an important cellular recycling process in eukaryotes for cell survival and maintenance. In normal physiological processes, autophagy can remove aged, aggregated and misfolded proteins, as well as damaged organelles, to regulate cell growth. Autophagy also plays an important role in the process of pathogen invasion. It was reported that *Helicobacter pylori* outer membrane vesicle induced host cell autophagy to limit the growth and survival of bacteria, which is an important antibacterial mechanism in gastric epithelial cells [11]. Certain pathogens can inhibit, block or even destroy the mechanism of autophagy to provide a stable environment for pathogen survival [12]. *Mycobacterium tuberculosis* can inhibit the process of autophagy to evade the clearance of host macrophages [13]. However, it is still unclear whether *C. parvum* can regulate the autophagy of host cells.

MicroRNAs, a group of non-coding RNAs that play a key role in host cellular response to various pathogens, including viruses, parasites and bacteria, are key factors in regulating autophagy [14]. MiR-30a, miR-376b, and miR-519a are involved in regulating autophagy via Beclin1 signaling, and miR-375 regulates autophagy via ATG protein [15]. It has been shown that miR-30a-5p could regulate autophagy by targeting Beclin1 during porcine circovirus type 2 and duck enteritis virus infection [16, 17]. MiR-30a-3p enhanced the megaautophagy and autophagy of host cells to reduce the intracellular proliferation of *Leishmania donovani* [18]. Autophagy is also known to be regulated by complex signaling pathways [19]. MAPK signaling pathways could regulate autophagy in the process of various diseases. P38 MAPK could inhibit autophagy by phosphorylating ULK1 or activate chaperone-mediated autophagy by phosphorylating LAMP2A [20, 21]. Ganoderma applanatum polysaccharide could induce autophagy in MCF-7 cells by downregulating p-ERK or upregulating p-P38 and p-JNK [22]. In hepatocellular carcinoma cells, novel 2-phenyloxypyrimidine derivative enhanced autophagy by activating ERK signaling [23]. A previous study suggested that DUSP5, a negative regulator of MAPK, was the potential target of miR-26a, which is involved in autophagy regulation [24]. MiR-139-5p promoted autophagy through MAPK/NF-κB signaling pathway [25]. However, it is not clear whether miRNA and MAPK signaling pathways are involved in regulating autophagy in HCT-8 cells infected with *C. parvum*.

MiRNA and MAPK signaling pathways are important in the process of parasite infection. During *Leishmania* infection, miR-7, miR-223, miR-133a and miR-146b showed high expression [26]. *Toxoplasma gondii* infection upregulated miR-155-5p and miR-29c-3p, but downregulated miR-21-5p and miR-125b-5p [27]. The downregulation of miR-322 in response to *Trypanosoma cruzi* infection plays an important role in the invasion process [28]. Therefore, the abnormal expression of miRNA in parasite infection could be considered a disease diagnostic indicator. *Leishmania* and *T. cruzi* targeted MAPK pathways to regulate the host immune system and promoted its replication and survival in macrophages [29]. In biliary epithelial cells, the expression of the let-7 family miRNAs was decreased by *C. parvum* [30]. After *C. parvum* infection, 20 miRNAs of HCT-8 cells exhibited significant differential expression [31]. Muñoz et al. reported that *C. parvum* could activate ERK and P38 MAPK pathways [32]. However, the roles of miRNAs and MAPK signaling pathways have not been investigated in intestinal epithelial cells infected with *C. parvum*.

The present study was designed to elucidate the role of host cell autophagy in *C. parvum* infection and the regulatory mechanism of *C. parvum* on autophagy. The study might provide new intervention targets for the prevention and treatment of *C. parvum.*

## Methods

### Cells and parasites

HCT-8 cells were preserved and propagated in our laboratory. HCT-8 cells were cultured with RPMI 1640 medium (Biological Industries, Israel) containing 10% FBS and 1% penicillin streptomycin (Biological Industries, Israel) at 37 °C in 5% CO_2_. When grown to 70–80% confluence, the cells were subjected to passaging or plating.

*Cryptosporidium parvum* (gene subtype: IIa A15G2R1) was preserved and propagated in our laboratory. *Cryptosporidium* oocysts, originally obtained from our laboratory, were used to infect 5-day-old Holstein calves (1×10^8^/animal). Following the onset of oocyst shedding, feces were collected daily, mixed with an equal volume of 5% potassium dichromate and stored at 4 °C. Oocyst isolation was through discontinuous sucrose gradients according to previous methods [33]. For purification of sporozoites, oocysts were resuspended in 0.8% sodium taurocholate (Sigma, MO, USA) with 2.5% trypsin (BOSTER, Wuhan, China), then incubated at 37 °C for 30 min, washed three times in PBS and resuspended in RPMI 1640 medium with 2% FBS and 1% penicillin-streptomycin. The excystation rate of sporozoites is about 70% using a cell counting plate, and the number of oocysts required is calculated as a ratio of 1:2 of sporozoites to HCT-8 cells.

### MiRNA detection

HCT-8 cells (2.5 × 10^6^ cells/well) were stimulated with sporozoite at a ratio of 1:2 (sporozoite:cell). After stimulation with sporozoites for 2 h, HCT-8 cells were thoroughly rinsed with fresh medium and cultured at 37 °C. Then, the cells were collected at 12 h, 18 h and 24 h post-sporozoite stimulation. Total RNAs were extracted from HCT-8 cells using TRIZOL reagent (Tiangen Biotech, Beijing, China) including chloroform extraction and isopropanol precipitation. The cDNA was constituted from total RNA using specific miRNA reverse transcription primers (miR-23a, miR-375, miR-30a and miR-26a, Ruibobio, Guangzhou, China) according to the manufacturer’s instructions (PrimeScript^™^ RT Master Mix, TaKaRa, China).

MiR-26a-mimic, miR-30a-mimic or ssRNA (negative control) was transfected into HCT-8 cells for 24 h; then, the cells were stimulated with sporozoite (sporozoite:cell = 1:2) for 12 h. Total RNAs were extracted from HCT-8 cells, and cDNA was synthesized from total RNA using specific miRNA reverse transcription primers according to the manufacturer’s protocols.

The qPCR reaction components are as follows: FastStart Universal SYBR Green Master 10 μl, specific quantitative real-time PCR (qPCR) primers (miR-23a, miR-375, miR-30a and miR-26a) 0.8 μl, cDNA 5 μl and ddH_2_O 4.2 μl. The qPCR was performed using the FastStart Universal SYBR Green Master (ROX) (Roche, Swiss) with the following conditions: an initial denaturation step at 94 °C for 10 min followed by 40 cycles of 94 °C for 20 s and 60 °C for 1 min using qTOWER2.0 (Analytik Jena AG, Germany).

### Transfection

HCT-8 cells were seeded in a 24-well plate at 5×10^5^ cells per well or 2×10^6^ cells per well in a 6-well plate with RPMI 1640 medium supplemented with 10% FBS (BI, Israel). The cells were transfected with miR-26a-mimic/inhibitor and miR-30a-mimic/inhibitor (Ruibobio, Guangzhou, China) (10 μl each well in 6-well plates and 2 μl each well in 24-well plates) or EGFP-LC3 (8 μg each well in 24-well plates) using Lipofectamine 2000 transfection reagent (Invitrogen, USA). Then, the cells were stimulated with sporozoites (sporozoite:cell = 1:2) in RPMI 1640 medium containing 2% FBS.

### Fluorescent microscopy detection

HCT-8 cells transfected with EGFP-LC3 were cultured in 24-well plates containing glass slides (2 × 10^5^ cells/well). At 24 h post-transfection, the miR-26a-mimic or miR-30a-inhibitor was transfected into the cells; cells were cultured with ERK inhibitor (LY3214996, 1 μM; Selleckchem, USA), P38 inhibitor (SB203580, 5 μM; Selleckchem, USA) or Rapamycin (1 μM, Selleckchem, USA) for 1 h. The cells were incubated with sporozoites (sporozoite:cell = 1:2) for 12 h before harvesting. Cells on glass slides were washed with PBS three times (5 min for each time), fixed with 4% paraformaldehyde for 15 min at room temperature (RT) and treated with 0.25% TritonX-100 (Life Technologies Corporation, USA) for 20 min at RT. Then, the cells were blocked with 3% Bovine Serum Albumin (Boster Biological Technology, USA) in PBS for 30 min at RT and incubated with a 1:100 dilution of the *C. parvum* virus capsid antibody (prepared in our laboratory) overnight at 4 °C. The cells were washed and incubated with CoraLite594 secondary antibody (Proteintech, Wuhan, China) for 1 h at RT. At last, the cells were sealed by Antifade Mounting Medium (BOSTER Biological Technology, USA) with 0.1% DAPI (Thermo Science, Waltham, MA) and observed under a laser scanning microscope (Zeiss LSM710, Germany).

### Western blotting

HCT-8 cells were stimulated with sporozoite at a ratio of 1:2 (sporozoite:cell). After stimulation with sporozoite for 2 h, HCT-8 cells were thoroughly rinsed with fresh medium, cultured and collected at 0 h, 2 h, 4 h, 8 h, 12 h, 18 h, and 24 h. Cells were pretreated with ERK inhibitor and P38 inhibitor for 1 h and stimulated with sporozoite (sporozoite:cell = 1:2) for 12 h; miR-26a-mimic, miR-26a-inhibitor, miR-30a-mimic, miR-30a-inhibitor and ssRNA (negative control) (Ribobio, Guangzhou, China) were transfected into HCT-8 cells for 24 h, and the cells were stimulated with sporozoite (sporozoite:cell = 1:2) for 12 h.

All the samples were lysed by the Whole Cell Lysis Assay (KeyGENBioTECH, Jiangsu, China) to extract proteins, and the concentration was determined by the Pierce BCA Assay (Thermo Science, Waltham, MA). Equal amounts of protein were resolved to either 12% SDS-PAGE and transferred onto PVDF membranes. Membranes were blocked in 5% skimmed milk (solution TBST) for 2 h and then incubated overnight with primary rabbit antibodies anti-phospho-p44/42 MAPK (Thr202/Tyr204), p44/42 MAPK (ERK1/2), anti-phospho-P38 MAPK (Thr180/Tyr182), anti-P38 MAPK, anti-phospho-SAPK/JNK (Thr183/Tyr185), anti-SAPK/JNK, anti-p62, anti-GAPDH, anti-Beclin1 and anti-LC3 (1:1000; Cell Signaling Technology, MA, USA). After washing, membranes were incubated with horseradish peroxidase-conjugated secondary antibody (1:5000; Proteintech, Wuhan, China) for 1 h at RT. Protein bands were visualized by chemiluminescent detection (GE Healthcare Life Sciences, USA). Quantification of western blotting bands was performed using Image J software (NIH, USA) analysis.

### Cells and parasite growth detection

HCT-8 cells were seeded in six-well plates (2 × 10^6^ cells/well). The cells were divided into two groups. For one group, miR-26a-mimic, miR-26a-inhibitor, miR-30a-mimic, miR-30a-inhibitor and ssRNA (negative control) were transfected into the cells for 24 h and stimulated with sporozoites (sporozoite:cell = 1:2) for 24 h. For the other group, cells were treated with rapamycin (1 μM), 3-MA (1 mM, Selleckchem, USA), LY3214996 or SB203580 for 1 h before being challenged with sporozoites (sporozoite:cell = 1:2) for 24 h.

The cells were collected, and the total RNA was extracted using TRIZOL reagent (Tiangen Biotech, Beijing, China) including chloroform extraction and isopropanol precipitation. Total RNA was reverse transcribed into cDNA using the RT Reagent Kit with gDNA Eraser (Takara, Beijing, China). The proliferation of *C. parvum* was examined by qPCR. The qPCR reaction was as follows: SYBR Green Master Mix 12.5 μl, primers 1 μl, cDNA 2 μl and ddH_2_O 8.5 μl. The primers for qPCR were as follows: Cp18S-1604F (5’-CCT ACG GAA ACC TTG TTA CGA-3’) and Cp18S-1943R (5′-AGT TTT AGG CAA TAA CAG GTC-3′) (GenBank accession number: NC_006986.1) (*C. parvum* 18S rRNA) and Hs18S-1246F (5′-ACT CAA CAC GGG AAA CCT CAC-3′) and Hs18S-1645R (5′-AGC TTA TGA CCC GCA CTT ACT GG-3′) (GenBank accession number: NR_003286) (human 18S rRNA). PCR was performed on a qTOWER 2.0 (Analytik Jena, Germany) with the following program: 95 °C for 3 min, followed by 40 cycles of 95 °C for 30 s and 60 °C for 30 s, with a final step at 70 °C to 95 °C for melting curves.

### Statistical analysis

Data were expressed as mean ± SD. Data were analyzed using one-way ANOVA with the Tukey-Kramer post hoc test or two-way ANOVA assay with the Bonferroni test. All graphs were generated using GraphPad Prism 5 (GraphPad Software, CA, USA). *P* < 0.05 was considered significant (*P < 0.05, **P < 0.01, ***P < 0.001). *P* ≥ 0.05 was considered not significant.

## Results

### Autophagy occurred in HCT-8 cells infected with C. parvum

**Fig. 1:**
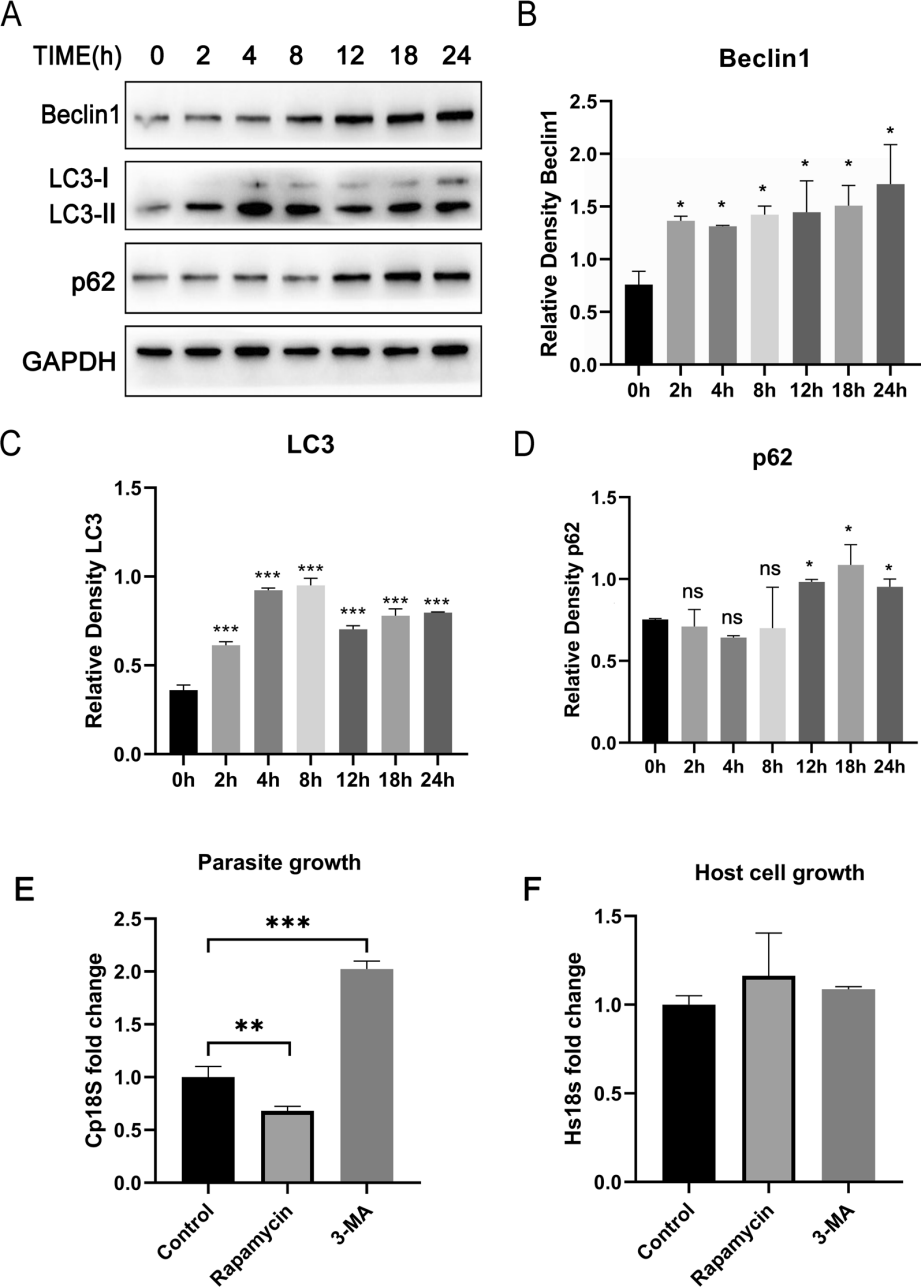
Autophagy occurred in HCT-8 cells infected with *C. parvum* HCT-8 cells, which were infected with *C. parvum* sporozoites for 0–24 h. The cellular samples were then analyzed by western blotting with rabbit anti-LC3, anti-Beclin1, anti-p62 and anti-GAPDH antibodies (**A**). The results were further analyzed by grayscale analysis from four independent experiments (**B**–**D**). HCT-8 cells were pretreated with rapamycin (1 μM) and 3-MA (1 mM). Parasite numbers (**E**) and cell numbers (**F**) were quantified by using qPCR assays at 24 h post-transfection infected with *C. parvum* sporozoites. A one-way ANOVA assay with the Tukey-Kramer post hoc test was used for analyzing data. Data are expressed as the mean ± SD from three independent experiments (*P < 0.05, **P < 0.01, ***P < 0.001, ns = no significant differences)

To confirm whether autophagy occurred in HCT-8 cells infected with *C. parvum*, the expression levels of Beclin1, LC3 and p62 were detected using western blotting. The results showed that the expression levels of Beclin1 gradually increased from 0 to 24 h post-*C. parvum* infection. The expression levels of LC3 increased gradually from 0 to 8 h, downregulated at 12 h and increased again from 18 to 24 h post-*C. parvum* infection. The expression levels of p62 showed no change from 0 to 8 h but increased after 12 h post-*C. parvum* infection. The results indicated that *C. parvum* induced autophagy in HCT-8 cells within 8 h, but inhibited autophagy from 12 to 24 h (Fig. 1A–D). To further detect cellular autophagy in *C. parvum*-stimulated HCT-8 cells, the accumulation of autophagosomes was examined. The results showed that *C. parvum* caused the accumulation of LC3 in HCT-8 cells (Additional file 1: Fig. S1). To investigate the roles of autophagy in *C. parvum* proliferation, the inhibitor (3-MA) and inducer (Rapamycin) of autophagy were used in HCT-8 cells infected with *C. parvum* (Fig. 1E, F). Treatment with rapamycin or 3-MA did not influence the number of HCT-8 cells. The numbers of *C. parvum* were decreased in HCT-8 cells treated with rapamycin (0.68-fold) while increased in the cells treated with 3-MA (2.02-fold). The data suggested that host cell autophagy could initially inhibit *C. parvum* proliferation, while *C. parvum* inhibited autophagy from 12 to 24 h post-infection to promote parasite survival.

### *Cryptosporidium parvum* inhibited autophagy of HCT-8 cell by regulating miR-26a and miR-30a expression

**Fig. 2:**
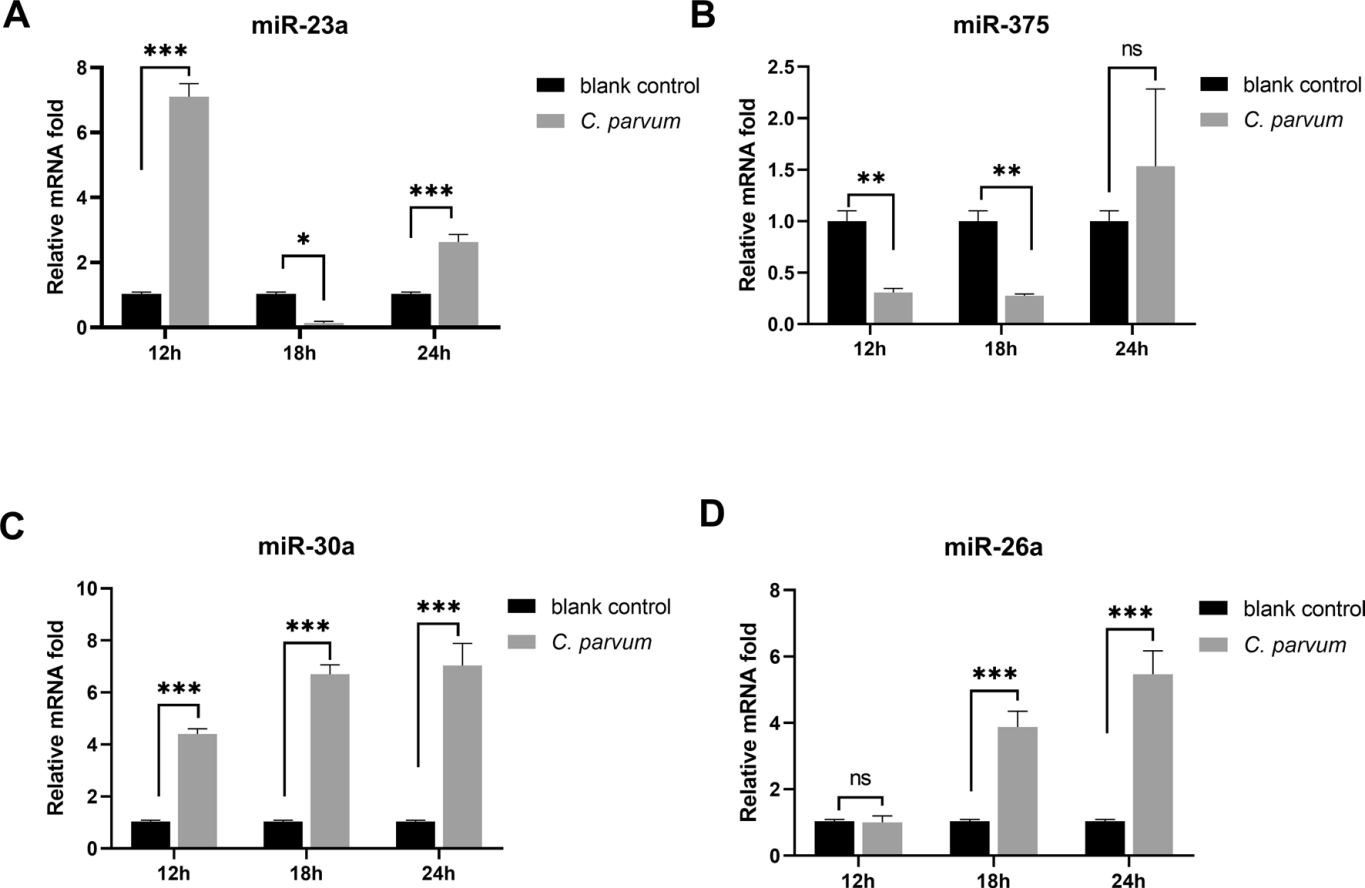
The expression of miR-23a, miR-375, miR-26a and miR-30a was detected in HCT-8 cells during *C. parvum* infection. HCT-8 cells were infected by *C. parvum* sporozoites. MiRNAs (miR-23a, miR-375, miR-26a and miR-30a) were examined at 12 h, 18 h and 24 h after *C. parvum* infection by qPCR. No infection group was the blank control. All these miRNAs were differentially expressed at different time points (**A**–**D**). Two-way ANOVA assay with Bonferroni test was used for analyzing data. Data are expressed as the mean ± SD from three independent experiments (*P < 0.05, **P < 0.01, ***P < 0.001, ns = no significant differences)

**Fig. 3:**
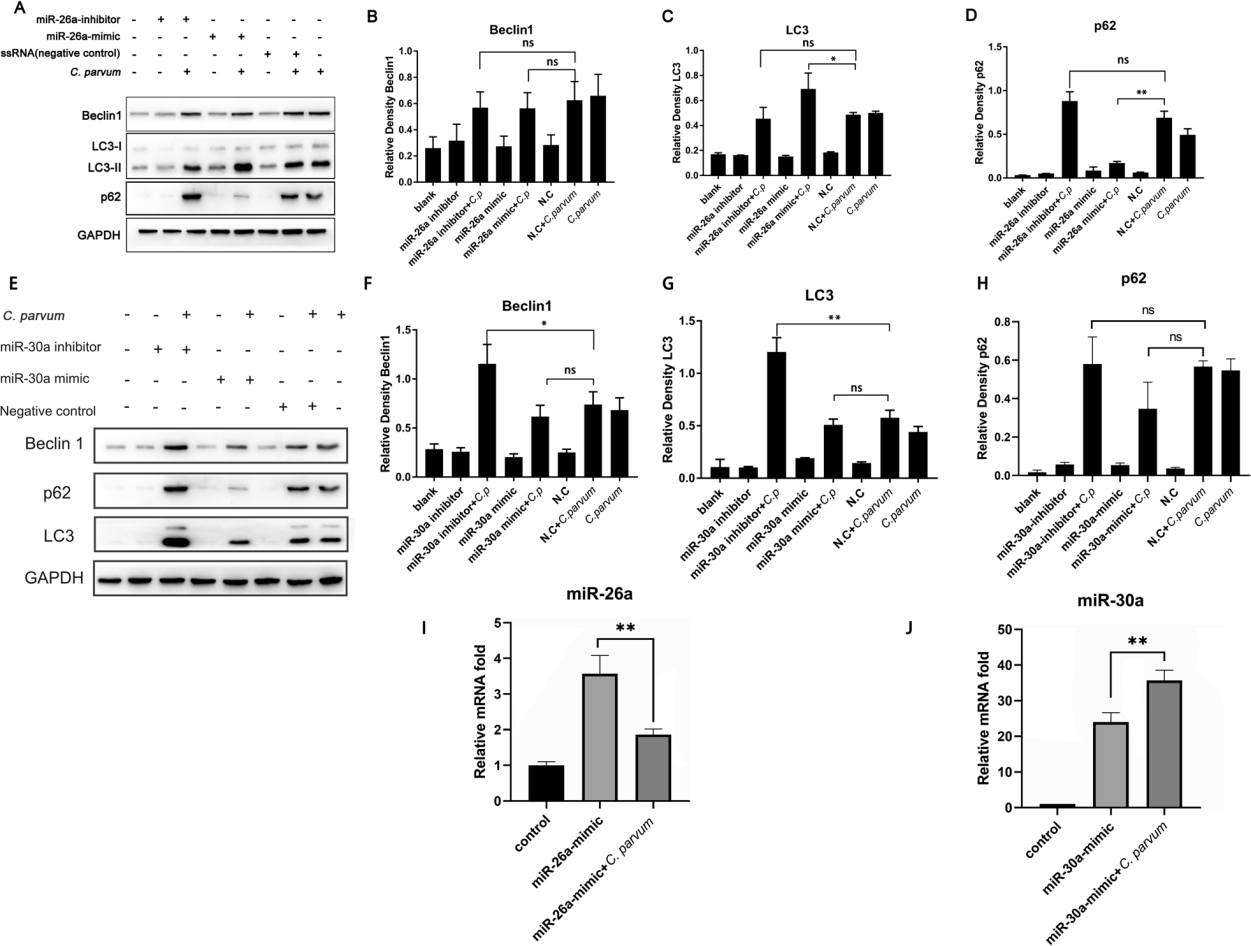
*Cryptosporidium parvum* inhibited autophagy of HCT-8 cell by regulating miR-26a and miR-30a expression. MiR-26a-mimic, miR-26a-inhibitor, miR-30a-mimic, miR-30a-inhibitor or ssRNA (negative control) was transfected into HCT-8 cells, and the cells were infected with *C. parvum* sporozoites at 24 h post-transfection. The protein samples were collected after 12 h infection and analyzed by western blotting with rabbit anti-LC3, anti-Beclin1, anti-p62 and anti-GAPDH antibodies (**A**, **E**). The results were further analyzed by grayscale analysis from three independent experiments (**B**–**D**, **F**–**H**). miR-26a-mimic, miR-30a-mimic or ssRNA (negative control) was transfected into HCT-8 cells, and the cells were infected with *C. parvum* sporozoites at 24 h post-transfection. The expression of miR-26a and miR-30a was detected using qPCR. A one-way ANOVA assay with Tukey-Kramer post hoc test was used for analyzing data. Data are expressed as the mean ± SD from three independent experiments (*P < 0.05, **P < 0.01, ns = no significant differences)

To clarify the mechanism by which *C. parvum* inhibited autophagy in HCT-8 cells, four miRNAs related to autophagy (miR-23a, miR-375, miR-26a and miR-30a) were examined at 12 h-24 h post-*C. parvum* infection using qPCR. The results revealed that *C. parvum* was involved in regulating the expression of the four miRNAs (Fig. 2A–D). However, only miR-26a and miR-30a expression was increased from 12 to 24 h post-*C. parvum* infection (Fig. 2C, D), which was consistent with the expression trend of autophagy proteins. These results suggested that *C. parvum*-regulated miR-26a and miR-30a might be involved in controlling autophagy in HCT-8 cells. To further confirm the roles of *C. parvum*-regulated miR-26a and miR-30a in autophagy, the expression levels of Beclin1, LC3 and p62 were detected in HCT-8 cells treated with the mimics or inhibitors of miR-26a and miR-30a. Although the miR-26a inhibitor resulted in a decrease in LC3 and Beclin1 expression while an increase in p62 expression, the difference was not significant. After the cells were transfected with miR-26a-mimic, no differential expression was observed for Beclin1. The expression of p62 was inhibited while LC3 was increased (Fig. 3A–D), which indicated that miR-26a could induce autophagy in HCT-8 cells infected with *C. parvum*. Moreover, the miR-30a inhibitor increased LC3 and Beclin1 expression (Fig. 3E–H), suggesting that miR-30a prevented autophagy in HCT-8 cells infected with *C. parvum*. In addition, treatment with miR-26a-mimic or miR-30a inhibitor promoted the accumulation of LC3 in HCT-8 cells (Additional file 1: Fig. S1). To further study the effect of *C. parvum* on miR-26a and miR-30a expression, miR-26a or miR-30a mimics were transfected into HCT-8 cells. *Cryptosporidium parvum* reduced the expression of miR-26a-mimic-induced miR-26a (0.47-fold) but increased the expression of miR-30a-mimic-promoted miR-30a (1.51-fold). These results suggested that *C. parvum* suppressed miR-26a expression and promoted miR-30a expression to downregulate autophagy levels.

### MiR-26a induced autophagy in HCT-8 cells by inhibiting ERK and P38 signaling

**Fig. 4:**
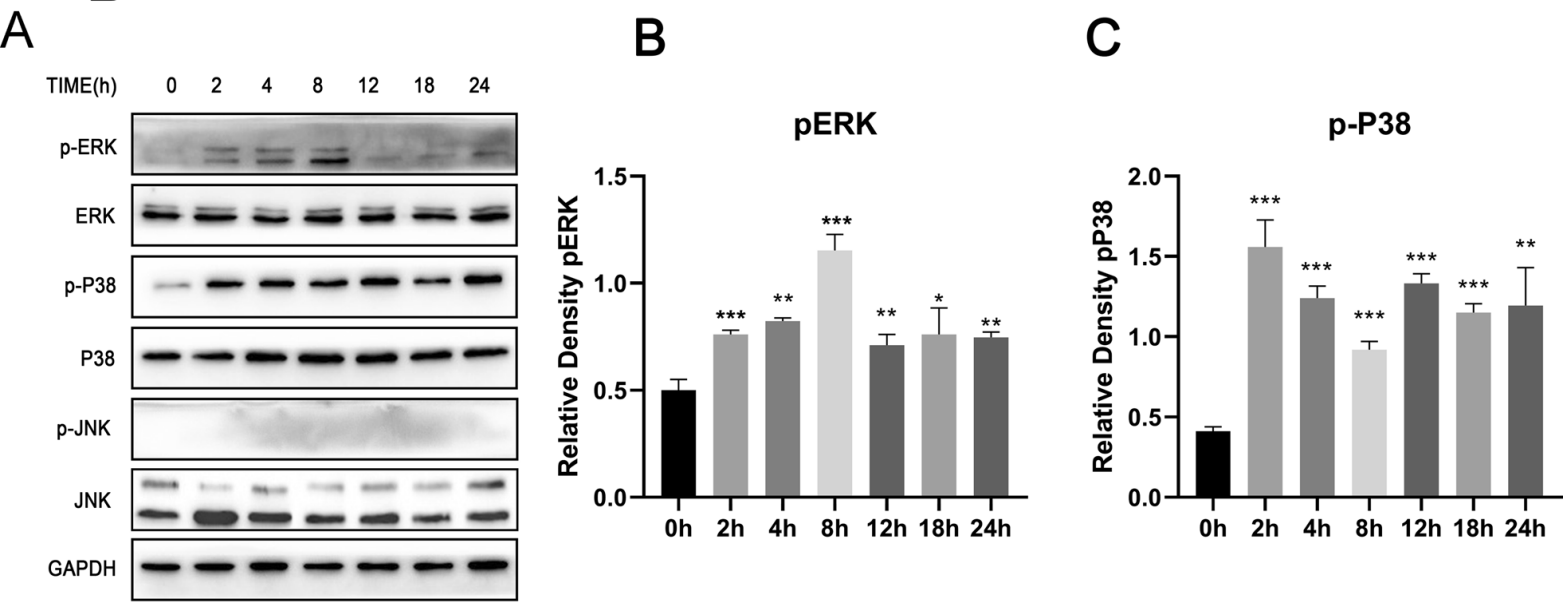
*Cryptosporidium parvum* activated MAPK signaling pathway in HCT-8 cells. HCT-8 cells were stimulated with *C. parvum* sporozoites from 0 to 24 h, cell lysates were used for western blotting using the rabbit anti-p-ERK, anti-ERK, anti-p-P38 MAPK, anti-P38 MAPK, anti-p-JNK and anti-JNK (**A**). The results were further analyzed by grayscale analysis from three independent experiments (**B**, **C**). A one-way ANOVA assay with Tukey-Kramer post hoc test was used for analyzing data. Data are expressed as the mean ± SD from three independent experiments (*P < 0.05, **P < 0.01, ***P < 0.001)

**Fig. 5:**
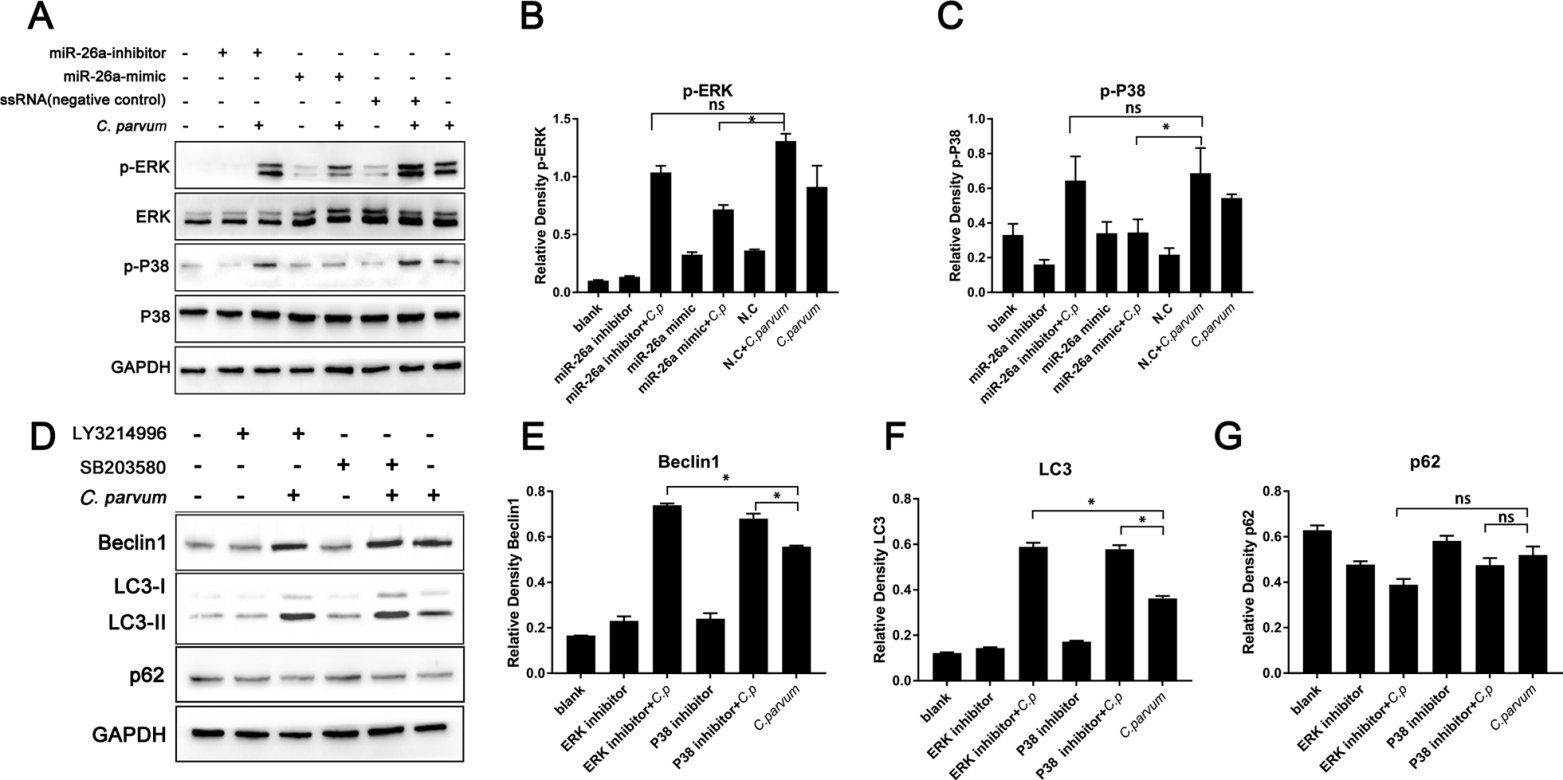
MiR-26a-induced autophagy in HCT-8 cells infected with *C. parvum* via p38 and ERK signaling. HCT-8 cells were transfected with miR-26a-mimic, miR-26a-inhibitor or ssRNA (negative control) and were infected with *C. parvum* sporozoites at 24 h post-transfection. Protein samples were collected at 12 h post infection and were analyzed by western blotting (**A**). The results were further analyzed by grayscale analysis from three independent experiments (**B**, **C**). HCT-8 cells were treated with ERK inhibitor LY3214996 (1 μM) and P38 inhibitor SB203580 (5 μM) for 1 h and then were infected with *C. parvum* sporozoites. The protein samples were collected at 12 h post infection and were detected by western blotting (**D**). The results were further analyzed by grayscale analysis from three independent experiments (**E**–**G**). A one-way ANOVA assay with Tukey-Kramer post hoc test was used for analyzing data. Data are expressed as the mean ± SD from three independent experiments (*P < 0.05, ns = no significant differences)

Previous studies suggest that miR-30a can directly target autophagy proteins [34]. To further explore the specific mechanism of miR-26a-regulated autophagy, the phosphorylation of MAPK signaling in HCT-8 cells infected by *C. parvum* was detected using western blotting. The results showed that *C. parvum* activated ERK and P38 MAPK signaling pathways. The phosphorylation levels of ERK gradually increased from 0 to 8 h but were not increased so high at 12 h. The phosphorylation levels of P38 MAPK were elevated from 2 to 24 h post-*C. parvum* infection. However, no phosphorylated JNK was detectable in HCT-8 cells (Fig. 4A–C). Next, the roles of miR-26a in the phosphorylation of ERK and P38 MAPK were analyzed. *Cryptosporidium parvum*-induced phosphorylation of ERK and P38 MAPK was significantly inhibited in HCT-8 cells by miR-26a-mimic (Fig. 5A–C). However, the phosphorylation of P38 and ERK was not significantly changed in HCT-8 cells treated with the miR-26a inhibitor. Subsequently, the relationship between ERK/P38 pathways and autophagy was examined. The results revealed that treatment with ERK inhibitor LY3214996 or P38 inhibitor SB203580 promoted the production of Beclin1 or LC3 and the accumulation of LC3 in HCT-8 cells (Fig. 5D–F, Additional file 1: Fig. S1). However, the production of p62 was not significantly changed (Fig. 5G). These data indicated that miR-26a could induce autophagy by inhibiting ERK and P38 signaling in HCT-8 cells infected with *C. parvum*.

### MiR-26a, miR-30a and ERK/P38 signaling regulated the proliferation of *C. parvum* in HCT-8 cells

**Fig. 6:**
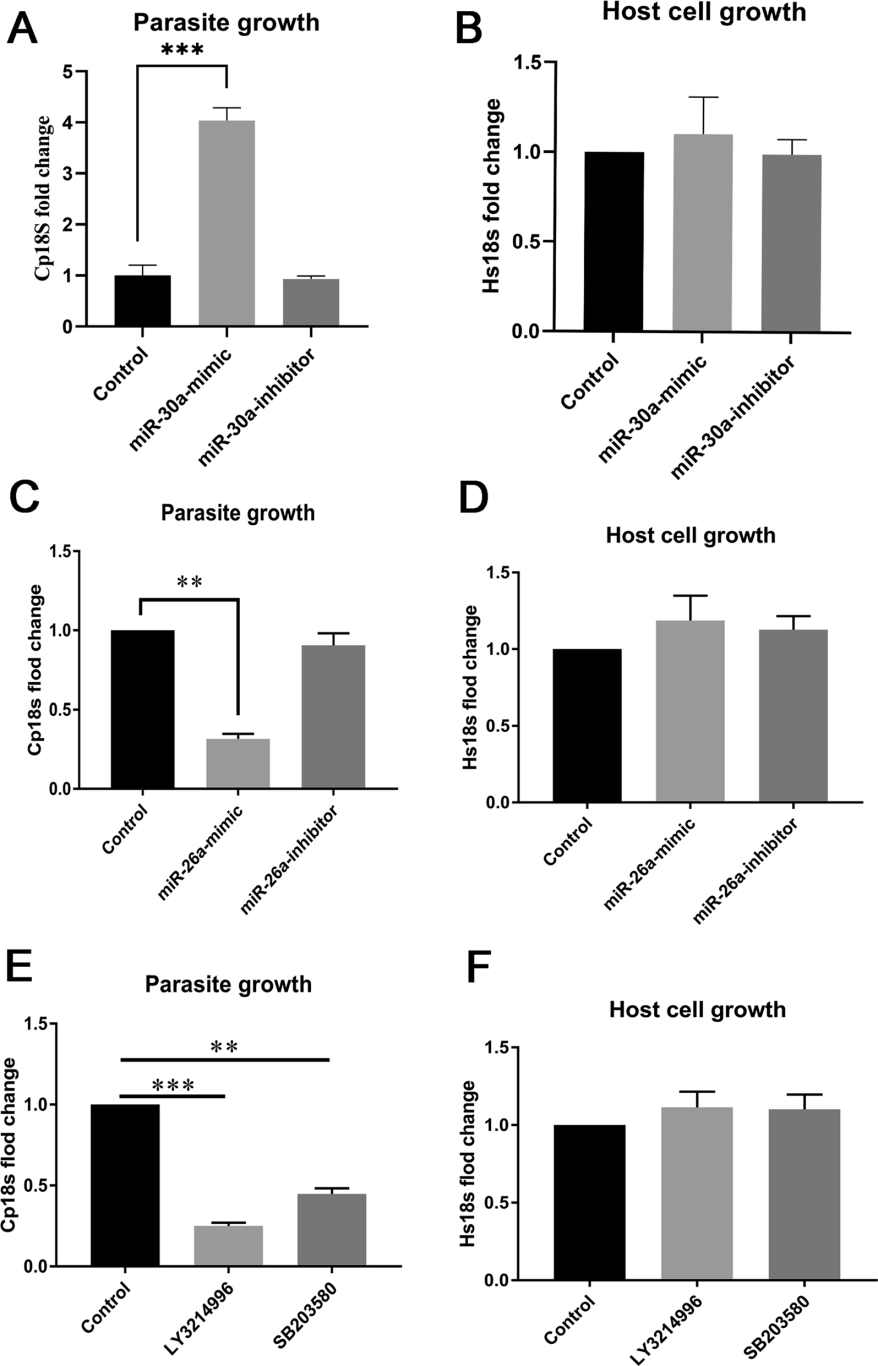
MiR-26a, miR-30a, ERK signaling and P38 signaling regulated *C. parvum* proliferation in HCT-8 cells. HCT-8 cells were transfected with miR-30a-mimic, miR-30a-inhibitor, miR-26a-mimic or miR-26a-inhibitor and were infected with *C. parvum* sporozoites for 24 h. Parasite numbers (**A**, **C**) and cell numbers (**B**, **D**) were quantified by using qPCR assays. HCT-8 cells were incubated with ERK inhibitor LY3214996 (1 μM) or P38 inhibitor SB203580 (5 μM) for 1 h before being challenged with *C. parvum* sporozoites for 24 h. Parasite numbers (**E**) and cell numbers (**F**) were quantified by using qPCR assays. A one-way ANOVA assay with the Tukey-Kramer post hoc test was used for analyzing data. Data are expressed as the mean ± SD from three independent experiments (*P < 0.05, **P < 0.01, ***P < 0.001)

To investigate the roles of miR-26a, miR-30a and ERK/P38 signaling in *C. parvum* proliferation, miR-26a-mimic or miR-26a-inhibitor, miR-30a-mimic or miR-30a-inhibitor, ERK inhibitor LY3214996 and P38 inhibitor SB203580 were used. Treatment with these inducers or inhibitors did not influence the number of HCT-8 cells (Fig. 6B, D, F). Transfection with miR-30a-mimic increased the number of *C. parvum* (4.02-fold) and transfection with miR-26a-mimic decreased the number of *C. parvum* (0.32-fold), whereas treatment with miR-26a-inhibitor and miR-30a inhibitor did not influence the number of *C. parvum* (Fig. 6A, C). Treatments with LY3214996 or SB203580 reduced the number of *C. parvum* to 0.25 times and 0.45 times compared with the control group (Fig. 6E). These results suggested that miR-26a inhibited while miR-30a or ERK/P38 signaling promoted *C. parvum* proliferation in HCT-8 cells.

## Discussion

Autophagy degrades lysosomes and recycles cytosolic components to keep cells homeostatic and is also involved in the capture and elimination of parasites. Treatment with DHA (an autophagy inducer) reduced the number of *T. gondii* in macrophages [35]. Rapamycin reduced the heart damage induced by *T. cruzi* by promoting cell autophagy [36]. Similarly, the present study revealed that promoting HCT-8 cell autophagy inhibited the proliferation of *C. parvum*. However, parasites are also able to inhibit autophagy or induce incomplete autophagy, which provides favorable conditions for parasite growth. *Trypanosoma cruzi* induced incomplete autophagy of cells, which was manifested in increased accumulation of LC3 and p62 [37]. *Toxoplasma gondii* prevented autophagy-mediated clearance by activating host EGFR signaling [38]. Our study showed that autophagy occurred initially in HCT-8 cells, which was inhibited from 12 to 24 h post-*C. parvum* infection to benefit parasite survival.

Several microRNAs were involved in response to *C. parvum* infection. *Cryptosporidium parvum* upregulated miR-942-5p expression and downregulated miR-181d expression in HCT-8 cells through TLR2/TLR4-NF-κB signaling [39, 40]. Twenty miRNAs, which are involved in cell apoptosis and immune responses, were differentially expressed in human intestinal epithelial cells after *C. parvum* infection [31]. We speculated that *C. parvum* regulated autophagy in HCT-8 cells via inducing miRNA expression. Thus, miR-26a, miR-30a, miR-23a and miR-375, which were related to the regulation of autophagy, were measured in HCT-8 cells stimulated with *C. parvum*. The present data showed that the expression of the four miRNAs was altered by *C. parvum* infection in HCT-8 cells. Only miR-26a and miR-30a expressions were consistent with the expression trend of autophagy proteins, suggesting that the two miRNAs might play major roles in regulating host cell autophagy.

A previous study revealed that lncRNA OTUD6B-AS1 promoted autophagy in triple-negative breast cancer cells by regulation of the miR-26a/MTDH pathway [41]. Rapamycin promoted autophagy dependent on the miR-26a-5p/DAPK1 pathway in glioma cells [42]. MiR-26a enhanced autophagy to protect against ethanol-induced acute liver injury [24]. Similarly, our study revealed that miR-26a promoted HCT-8 cell autophagy during *C. parvum* infection. However, MiR‑30a mitigated autophagy in renal ischemia/reperfusion injury [43]. C‑Myc promoted miR‑30a‑5p transcription to inhibit senescent cardiomyocyte autophagy [44]. In the present study, *C. parvum*-induced miR‑30a also inhibited HCT-8 cell autophagy. We found that *C. parvum* inhibited miR-26a-mimic-induced miR-26a expression and promoted miR-30a-mimic-induced miR-26a. HCT-8 cell autophagy was inhibited from 12 to 24 h post-*C. parvum* infection. Thus, these findings revealed that *C. parvum* inhibited miR-26a expression and promoted miR-30a expression to inhibit autophagy.

What could the mechanism of regulation autophagy by miR-26a and miR-30a be in HCT-8 cells infected with *C. parvum*? It is noted that miR-30a targeted Beclin-1 to inactivate autophagy in gastrointestinal stromal tumor cells [45]. miR-30a targeted ATG5 and attenuated autophagy in lung epithelial cells [34]. These results indicated that miR-30a could inhibit autophagy by targeting autophagy proteins, suggesting that *C. parvum*-induced miR-30a directly prevented autophagy in HCT-8 cells.

However, miR-26a induced autophagy in triple-negative breast cancer cells via the MTDH pathway and miR-26a induced autophagy in glioma cells via the DAPK1 pathway [41, 42], suggesting that miR-26a might indirectly inhibit autophagy. Meanwhile, a previous study showed that miR-26a might upregulate the expression of HIF-1a by activating the ERK pathway in BMECs [46]. MiR-26a overexpression or knockdown modulated the activity of P38 MAPK in EPCs. Interestingly, we also found that *C. parvum* infection induced the phosphorylation of ERK and P38 in HCT-8 cells, and the phosphorylation levels of ERK and P38 were reduced in miR-26a-mimic-treated cells infected with *C. parvum*. The combined treatment of BCL2L1 inhibitor and BCL2 inhibitor induced autophagy via NOX4/ROS/P38 MAPK axis [47]. However, P38 signaling may inhibit hunger-induced autophagy through the Atg5 protein [48]. Mouse embryonic palatal cells triggered autophagy through the ROS/ERK signaling pathway to avoid cell damage caused by nicotine [49]. Sulfated alginate oligosaccharide exerted autophagy induction by inactivating MEK1/ERK/mTOR signaling in osteosarcoma [50]. Ganoderma applanatum polysaccharide could promote the autophagy of MCF-7 cells by upregulating ERK phosphorylation or downregulating P38 MAPK and JNK phosphorylation [22]. These data revealed the roles of P38 or ERK signaling in autophagy were different in different cells. In this study, *C. parvum*-caused autophagy levels were increased in HCT-8 cells after treatment with P38 or ERK inhibitor, and miR-26a induced autophagy by inhibiting P38 and ERK signaling.

MiR-26a targeted EphA2 to resist intracellular *Listeria monocytogenes* in macrophages [51]. MiR-26a inhibited feline herpesvirus 1 replication by targeting SOCS5 and promoting type I interferon signaling [52]. Similarly, promoting miR-26a expression inhibited *C. parvum* proliferation. MiR-30a modulates type I interferon responses to facilitate coxsackievirus b3 replication by targeting tripartite motif protein 25, whereas miR-30a-5p can inhibit duck enteritis virus replication by reducing autophagy by targeting Beclin-1 [17]. In this study, promoting miR-30a expression increased *C. parvum* proliferation by inhibiting autophagy. Besides, ERK and P38 MAPK were involved in mediating NET formation during *C. parvum* invasion [32]. P38 signaling promoted *N. caninum* proliferation, but ERK signaling inhibited *N. caninum* infection in macrophages [53, 54]. However, the roles of the MAPK signaling pathway in host defense against *C. parvum* in intestinal epithelial cells have not been investigated. Our research further complemented the functional role of ERK and P38 in promoting *C. parvum* proliferation via suppressing autophagy.

## Conclusions

Taken together, the present study indicated that autophagy occurred and inhibited *C. parvum* proliferation in HCT-8 cells, whereas *C. parvum* inhibited miR-26a expression and promoted miR-30a expression to inhibit autophagy for parasite survival. Besides, miR-26a could induce autophagy by inhibiting ERK and P38 signaling in HCT-8 cells. These findings indicated that the downregulation of miR-26a, upregulation of miR-30a, and ERK and P38 MAPK activation in host cells induced by *C. parvum*-inhibited autophagy, which was required for parasite survival. This provides novel therapeutic targets for *C. parvum* infection.

## Supplementary Information

**Additional file 1: Figure S1.** MiR-26a, miR-30a, ERK and P38 regulated the accumulation of the EGFP-LC3 in HCT8 cells. EGFP-LC3 and miR-26a-mimic or miR-30a-inhibitor were transfected into HCT-8 cells for 24 h, and then the cells were challenged with *C. parvum* sporozoites for 12 h. Besides, after being transfected with EGFP-LC3 for 24 h, HCT-8 cells were incubated with ERK inhibitor LY3214996 (1 μM) or P38 inhibitor SB203580 (5 μM) for 1 h and were challenged with *C. parvum* sporozoites for 12 h. The accumulation of the EGFP-LC3 was observed by immunofluorescence. Red fluorescence = *C. parvum* (594), green fluorescence = LC3, and DAPI (blue) was used to stain nuclear DNA (Scale bar=5 µm).

**Additional file 2: Figure S2.** *Cryptosporidium parvum *and HCT-8 cells. **A** Purified oocysts. **B** Sporozoites. **C** HCT-8 cells. **D** HCT-8 cells were stimulated with sporozoite at a ratio of 1:2, and the cells were observed by immunofluorescence. The sporozoites were green (green arrow, *C. parvum* virus capsid antibody), and the nucleus was blue (blue arrow).

## Data Availability

All data generated or analyzed during this study are included in this published article.

## Abbreviations

HCT-8: Human colon adenocarcinoma HCT-8 cell line
miRNA: MicroRNA
MAPK: Mitogen-activated protein kinase
PBS: Phosphate balanced solution
qPCR: Quantitative real-time PCR
mRNA: Messenger ribonucleic acid
PVDF: Polyvinylidene fluoride
BSA: Albumin from bovine serum
DAPI: 4’,6-Diamidino-2-phenylindole
cDNA: Complementary deoxyribonucleic acid
SDS-PAGE: Sodium dodecyl sulfate-polyacrylamide gel electrophoresis
TBST: Tris-buffered saline with Tween-20
