# Supplementary figures and images for: Cryptosporidium parvum regulates HCT-8 cell autophagy to facilitate survival via inhibiting miR-26a and promoting miR-30a expression
Source: Parasit Vectors. 2022 Dec 15;15:470. doi: 10.1186/s13071-022-05606-y (PMC9756778; doi:10.1186/s13071-022-05606-y)

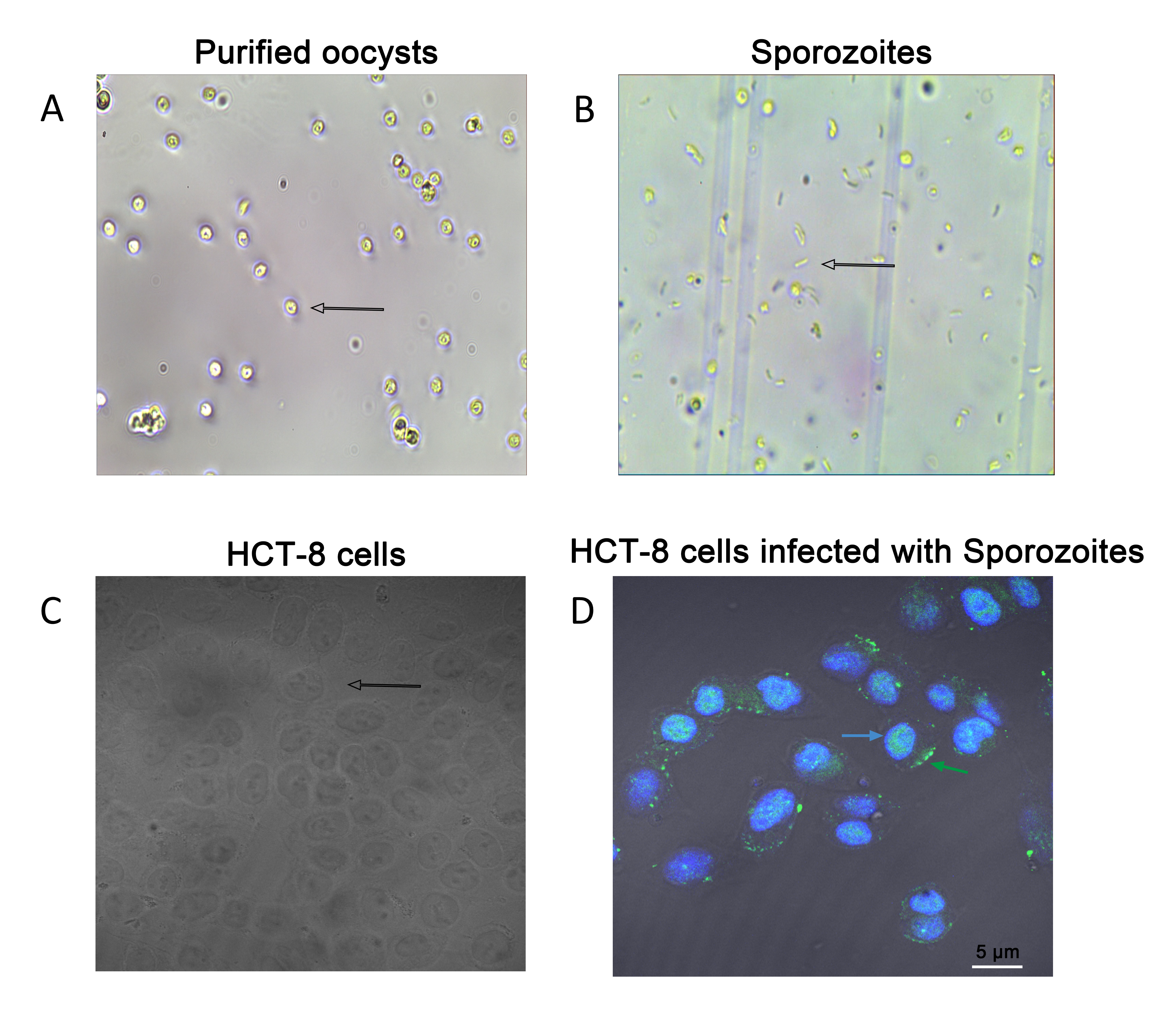

Supplement: Supplementary file 2 — Additional file 2: Figure S2. Cryptosporidium parvum and HCT-8 cells. A Purified oocysts. B Sporozoites. C HCT-8 cells. D HCT-8 cells were stimulated with sporozoite at a ratio of 1:2, and the cells were observed by immunofluorescence. The sporozoites were green (green arrow, C. parvum virus capsid antibody), and the nucleus was blue (blue arrow). [file 13071_2022_5606_MOESM2_ESM.tif]
